# Genetic risk for neurodevelopmental disorders as a potential factor affecting antipsychotic responsiveness in schizophrenia: a postmortem brain study

**DOI:** 10.3389/fpsyt.2026.1812198

**Published:** 2026-04-27

**Authors:** Kazusa Miyahara, Mizuki Hino, Risa Shishido, Atsuko Nagaoka, Hideomi Hamasaki, Akiyoshi Kakita, Hiroaki Tomita, Yasuto Kunii

**Affiliations:** 1 Department of Disaster Psychiatry, International Research Institute of Disaster Science, Tohoku University, Sendai, Japan; 2 Department of Neuropsychiatry, School of Medicine, Fukushima Medical University, Fukushima, Japan; 3 Department of Psychiatry, Tohoku University Hospital, Miyagi, Japan; 4 Department of Pathology, Brain Research Institute, Niigata University, Niigata, Japan; 5 Department of Psychiatry, Graduate School of Medicine, Tohoku University, Miyagi, Japan

**Keywords:** attention deficit hyperactivity disorder, autism spectrum disorder, polygenic risk score, schizophrenia, treatment resistance

## Abstract

**Introduction:**

Schizophrenia (SCZ) is a highly heritable neuropsychiatric disorder. Its genomic architecture reportedly overlaps with that of neurodevelopmental disorders, such as attention deficit hyperactivity disorder (ADHD) and autism spectrum disorder (ASD). However, the effect of genomic risk for ADHD and ASD on SCZ symptoms remains unclear.

**Method:**

We obtained genome-wide association study (GWAS) data from the postmortem brains of 24 patients with SCZ and 48 controls and calculated the polygenic risk scores (PRSs) for ADHD (ADHD-PRS) and ASD (ASD-PRS) using publicly available GWAS data. For 19 patients with SCZ whose antemortem clinical information was available, we conducted correlation analyses between PRSs, severity of SCZ symptoms, and the antipsychotic responsiveness score (ARS). Additionally, we divided the patients into two subgroups based on ADHD-PRS (high and low ADHD-PRS groups) and performed exploratory gene expression analyses and subsequent pathway analysis in the prefrontal cortex.

**Results:**

The ARS of positive symptoms (ARS-PS) demonstrated a suggestive negative correlation with ADHD-PRS and a positive correlation with ASD-PRS although these associations did not survive multiple testing correction. No correlation was observed between the ARS of general psychopathology or the ARS of negative symptoms and either ADHD-PRS or ASD-PRS. Gene expression analysis identified 1,773 DEGs, including neuropsychiatric disorder-related genes including *CHRNB2*. These DEGs were enriched in pathways associated with the neuronal system and mitochondrial function.

**Discussion:**

Our findings suggest that the genomic risk for neurodevelopmental disorders may affect the antipsychotic responsiveness of patients with schizophrenia and implicate translational alterations in potential marker molecules in this phenotype. Due to the limited sample size in the current study, further investigation on the large cohort is required to verify our exploratory findings.

## 1 Introduction

Schizophrenia (SCZ) is a major psychiatric disorder with a relatively high heritability rate of approximately 70% (1). Despite numerous molecular genetic studies, including genome-wide association studies (GWAS) (2), the molecular pathogenesis of SCZ remains unclear. Multiple antipsychotic drugs targeting various neurotransmission systems, including dopamine (3), have been developed and are widely used (4). Although these drugs are effective for some patients with SCZ, their effects vary widely among individuals. Approximately 30% of patients continue to experience persistent symptoms despite treatment with two or more antipsychotic drugs at adequate doses and durations, a condition defined as treatment-resistant SCZ (TRS) (5). The mechanisms involved in the responsiveness to these antipsychotic drugs have not been elucidated.

Individuals with SCZ sometimes exhibit several commonalities with neurodevelopmental disorders, such as attention-deficit/hyperactivity disorder (ADHD) and autism spectrum disorder (ASD). ADHD, one of the most common neurodevelopmental disorders, is characterized by hyperactivity and difficulties in sustaining attention. Patients with SCZ also exhibit cognitive impairments (6) and emotional dysregulation (7) similar to those observed in ADHD, suggesting partial overlap in brain endophenotypes despite distinct molecular pathophysiology. Epidemiological studies indicate that children with ADHD are at increased risk of developing SCZ (8, 9) and that genetic ADHD risk elevates SCZ risk (10, 11). Similarly, several ASD-related symptoms, such as impairment in social communication, are also observed in SCZ. Patients with SCZ exhibit autistic traits, including impaired social communication and reduced emotional expression (12). Furthermore, individuals diagnosed with ASD in their childhood have a three- to six-fold higher risk of developing SCZ than those without the diagnosis (13, 14). The Positive and Negative Syndrome Scale (PANSS) is a widely used measure of SCZ symptom severity. The PANSS Autism Severity Score (PAUSS), a sub-domain of PANSS, was developed to assess ASD characteristics in patients with SCZ. Higher PAUSS scores are associated with poorer performance in terms of processing speed, attention, verbal memory, and social cognition (15). Additionally, higher PAUSSs predict poorer social relationships (16) in patients with SCZ. Moreover, a shared genetic architecture has been repeatedly pointed out between SCZ and neurodevelopmental disorders such as ADHD and ASD (17, 18). Kuramitsu et al. examined the effect of genetic risk for ADHD and ASD on SCZ symptoms using polygenic risk scores (PRSs) to differentiate between the two disorders, reporting an association between ADHD genetic risk and impaired working memory (19). These findings indicate commonalities between SCZ, ADHD, and ASD, suggesting that exploring neurodevelopmental traits in SCZ may provide important insights into its pathophysiology.

Emerging evidence also links ADHD and ASD to antipsychotic responsiveness in SCZ. For example, a positive genetic correlation was observed between TRS and ADHD (20), and patients with TRS exhibit more pronounced autistic traits than those in remission (21). However, it remains unclear whether genetic risks for ADHD and ASD are associated with clinical symptom scales or antipsychotic responsiveness in SCZ. Here, we aimed to investigate the relationship between the genetic risks for ADHD and ASD and the clinical phenotype of SCZ, including symptom severity and pharmacotherapeutic responsiveness to antipsychotic treatment, while also identifying the molecular basis in postmortem brains. The current study is pioneering in a small sample size that examined the influence of neurodevelopmental disorders’ genetic risk on the pathophysiology of SCZ through stratification based on PRSs.

To assess the genetic risk for ADHD and ASD, we calculated the PRSs for ADHD (ADHD-PRS) and ASD (ASD-PRS) using postmortem brain samples from 72 individuals, including 48 non-psychiatric controls and 24 patients with SCZ. We then examined associations between PRSs, clinical symptom severity, and antipsychotic responsiveness in 19 patients with available antemortem data. Finally, to explore molecular differences, patients with SCZ were classified into high and low ADHD-PRS subgroups, and gene expression profiles in the prefrontal cortex (PFC) were compared using RNA sequencing (RNA-seq), followed by pathway analysis.

## 2 Subjects and methods

### 2.1 Subjects

Postmortem brain samples from 24 patients with SCZ and 48 controls were obtained from the Tohoku Postmortem Brain Bank and DNA Bank for Psychiatric Research (https://www.fmu-bb.jp/english/index.htm), and the Brain Research Institute, Niigata University, as previously described (22, 23). Appropriate statistical analyses controlling confounders adjust differences between the two institutions as shown in the previous studies (24, 25). This study was approved by the ethics committees of Fukushima Medical University, Niigata University, and the Tohoku University Graduate School of Medicine. All procedures were performed after obtaining written informed consent from the next of kin. SCZ diagnosis was based on the criteria of the Diagnostic and Statistical Manual of Mental Disorders, Fourth or Fifth Edition (DSM-IV or 5). The Diagnostic Instrument for Brain Studies (DIBS) was employed to assess the antemortem symptoms of each patient with SCZ, recorded within 3 months before death (26–29). Symptoms were categorized into three subscales based on the PANSS: positive, negative, and general psychopathology (30). The scores were obtained at the time of the maximum total DIBS score and 3 months before death. The daily antipsychotic dosage for the 19 patients with SCZ (3 months before death) was expressed as the chlorpromazine equivalent dose (CP-eq). Responsiveness to antipsychotics was evaluated using the antipsychotic responsiveness score (ARS), calculated as the ratio of relative responsiveness to daily CP-eq. Relative responsiveness was calculated as the rate of improvement in each DIBS subscale score relative to the subscale score at its most severe point. None of the patients had taken clozapine.


$$(antipsychotic\;responsiveness\;score)=\frac{\left(change\;rate\;of\;the\;DIBS\;subscale\;score\right)}{\left(daily\;CP-eq\;at\;three\;months\;before\;death\right)}$$

$$(change\;rate\;of\;the\;DIBS\;subscale\;score)=\frac{\left(improvement\;of\;the\;DIBS\;subscale\;score\;from\;the\;most\;severe\right)}{\left(the\;DIBS\;subscale\;score\;when\;most\;severe\right)}$$

### 2.2 Genotyping, imputation, and PRS calculation

Genotyping, imputation, and PRS calculations were performed as previously described (23). Briefly, genomic DNA was extracted from frozen cerebellum or occipital cortex samples and genotyped using Infinium Human Exome-12 v1.2 and HumanCoreExome-24 v1.0 Beadchip on an iScan system (Illumina, Tokyo, Japan). Demographic details of all subjects are presented in **Supplementary Table 1**. The following criteria were used to select single nucleotide polymorphisms (SNPs): 1) located in the autosomal region, 2) call rate > 90%, and 3) not duplicated or ambiguous. Genotype imputation was performed on these SNPs using a Multi-ethnic Imputation System (https://misystem.cgm.ntu.edu.tw) (31) with the 1000 Genomes Project Phase 3 dataset of East Asian ancestry (32) as a reference panel. In the quality checks, SNPs that fulfilled any of the following criteria were excluded: 1) minor allele frequency < 0.01, and 2) deviation from Hardy–Weinberg equilibrium (*p* < 1.0 × 10^–5^), leaving 81,680,095 SNPs. Additionally, samples with total genotyping rates < 0.99 were excluded, resulting in 23 patients with SCZ and 47 controls. Since no samples demonstrated high relatedness (>0.2), all were retained for analysis. PRS was calculated using PLINK v1.9 (http://www.cog-genomics.org/plink/1.9) (33) and PRSice-2 (34). Discovery GWAS datasets were obtained from publicly available studies of European ancestry from the Psychiatric Genomics Consortium and the iPSYCH project for both ASD-PRS (35) and ADHD-PRS (36). Before calculating PRS, SNPs with low information scores (<0.8) were excluded and clumped based on a pairwise r^2^ threshold of 0.1 and a 200 kb window. PRSs were calculated at four *p*-value thresholds: 0.01, 0.03, 0.05, and 1.00.

### 2.3 Measurement of mRNA expression levels

RNA isolation and sequencing were performed as previously described (22, 23). Total RNA was extracted from the PFC of frozen brains using an AllPrep DNA/RNA Mini Kit (Qiagen, Tokyo, Japan). RNA purity was evaluated by the RNA integrity number (RIN) using an Agilent 2200 TapeStation (Agilent, Santa Clara, CA, USA). The poly (A) fraction was isolated from the total RNA. Base pairs (bp) of double-stranded (ds) complementary DNA (cDNA) were reverse-transcribed from the fragmented mRNA. The ds-cDNA fragments were processed to generate cDNA libraries, which were then subjected to paired-end 2 × 101 bp sequencing on the HiSeq 4000 platform (Illumina, Tokyo, Japan). Data were obtained from 19 patients with SCZ in the PFC, and no replicate experiments were conducted.

### 2.4 Statistical analyses

Potential confounders between the patients and controls included in the PRS analysis (**Supplementary Table 1**) were compared using the Fisher–Freeman–Halton exact test for categorical variables (sex) and Welch’s t-test for continuous variables (age). ADHD-PRS and ASD-PRS values were compared between the SCZ and control groups using Welch’s t-test. Spearman’s rank test was conducted to assess the relationship between antemortem clinical information (DIBS subscale and ARS) and PRS (ADHD-PRS and ASD-PRS), conducting the Benjamini–Hochberg procedure for multiple testing correction. Patients were then stratified into high and low ADHD-PRS subgroups based on the median ADHD-PRS. Demographic characteristics were compared between subgroups (**Table 1**) and between the SCZ and control groups. The ARS of positive symptoms (ARS-PS) was also compared between the two subgroups using Welch’s t-test. Differentially expressed genes (DEGs) between high and low ADHD-PRS subgroups were identified using generalized linear models in the edgeR software package (version 4.0.16) (37), setting sex and age (categorized into groups of 10 years) as covariates. To avoid overfitting due to too many covariates (38), we did not include other covariate such as postmortem interval and RIN as with a previous study on human postmortem brain (39). To avoid sex chromosome-related bias, only autosomal genes were analyzed. The expression ratio was calculated using the low ADHD-PRS group as the reference. The Benjamini–Hochberg procedure was applied for multiple testing correction, and genes with nominal *p* < 0.05 were considered DEGs. Functional enrichment of DEGs (nominal *p* < 0.05) was evaluated by canonical pathway analysis using Ingenuity Pathway Analysis (IPA). For all other analyses, *p* < 0.05 was considered statistically significant.

**Table 1:** Demographic data of patients with schizophrenia.

Category	Schizophrenia		*P*-value
	High ADHD-PRS	Low ADHD-PRS	
**Number of samples**	9	10	
**Sex**			1^a^
Male	6	7	
Female	3	3	
**Age at death (years)** **^b^**	70.3 ± 8.2	64.3 ± 11.2	0.20 ^c^
**RIN** **^b^**	6.5 ± 0.7	6.7 ± 0.5	0.60 ^c^
**DOI (years)** **^b^**	42.9 ± 7.4	40.6 ± 9.3	0.56 ^c^
**CP-eq (mg/day)** **^b^**	523.3 ± 491.5	714.5 ± 729.7	0.51 ^c^
**ADHD-PRS** **^b^**	-0.005 ± 2.1×10^-5^	-0.005 ± 2.1×10^-5^	8.8×10–^5^ ^c^
**ASD-PRS** **^b^**	-0.03 ± 8.6×10^-5^	-0.03 ± 5.5×10^-5^	0.82 ^c^
**DIBS**			
Positive symptoms scale	8.6 ± 6.6	9.6 ± 8.1	0.76 ^c^
Negative symptoms scale	2.8 ± 2.5	3.8 ± 2.0	0.40 ^c^
General psychopathology scale	3.2 ± 1.6	3.0 ± 1.7	0.77 ^c^

RIN, RNA integrity number; DOI, duration of illness; CP eq, chlorpromazine equivalent of antipsychotics; DIBS, Diagnostic Instrument for Brain Studies.

^a^ Fisher–Freeman–Halton exact test.

^b^ Data are reported as mean ± standard deviation.

^c^ Welch’s t-test.s

Bold text indicates main demographic category.

## 3 Results

### 3.1 Calculation of PRS

**Figure 1:**
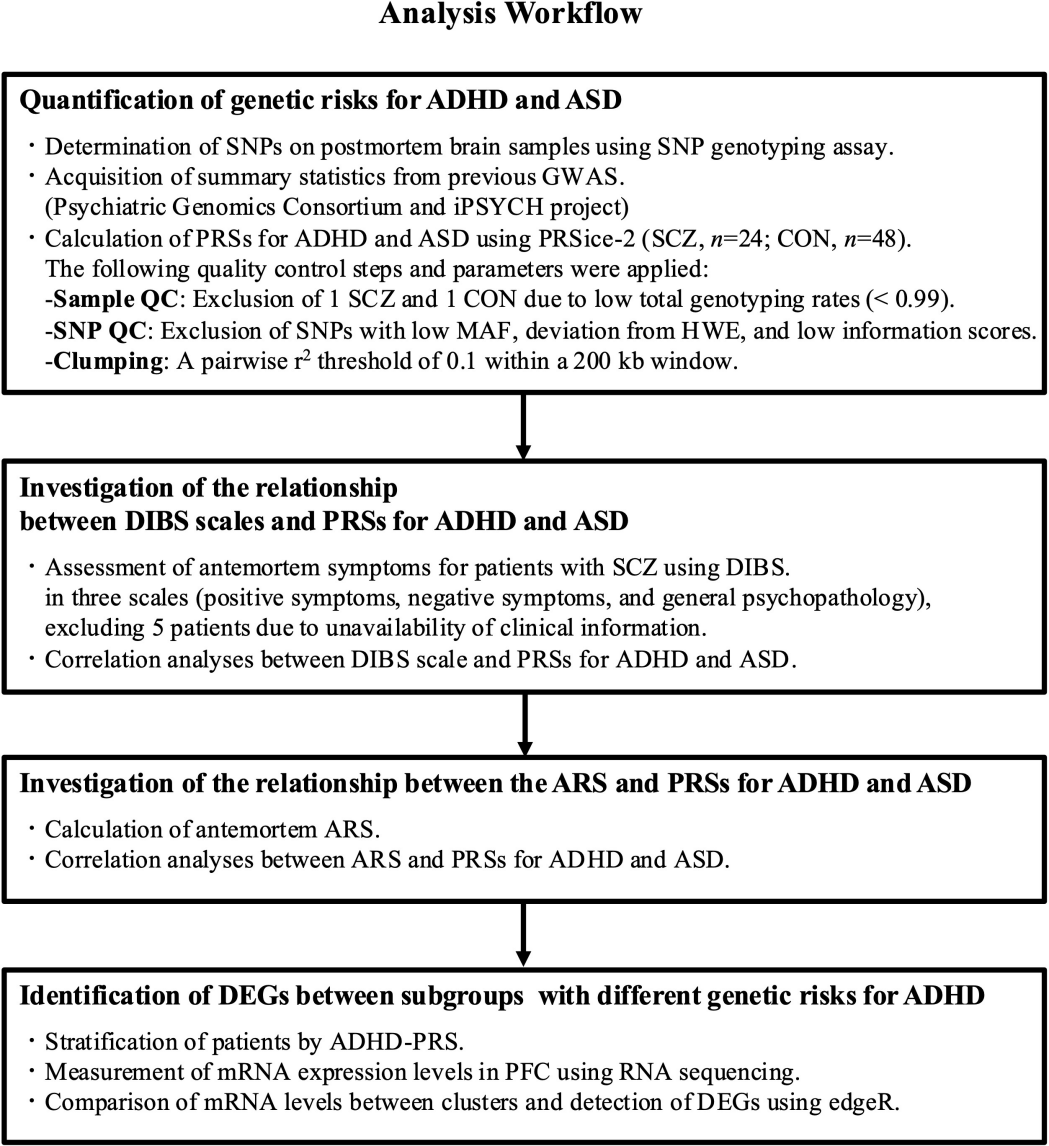
Analysis workflow. ADHD, attention-deficit/hyperactivity disorder; ARS, antipsychotic responsiveness score; ASD, autism spectrum disorder; CON, controls; DEG, differentially expressed genes; DIBS, The Diagnostic Instrument for Brain Studies; HWE, Hardy-Weinberg equilibrium; MAF, minor allele frequency; PFC, prefrontal cortex; PRS, polygenic risk score; QC, quality check; SCZ, schizophrenia.

The analysis workflow is shown in **Figure 1**. We calculated the PRS to assess the genetic risk for ADHD and ASD. The ADHD-PRS and ASD-PRS scores were calculated for 23 patients with SCZ and 47 controls across four different *p*-value thresholds. The highest variance explaining the onset risk of SCZ was observed at *p*-value thresholds of 0.03 for ADHD-PRS (**Supplementary Figure 1A**) and 0.01 for ASD-PRS (**Supplementary Figure 1B**). Therefore, we adopted the PRS calculated under these *p*-value thresholds for subsequent analyses. ADHD-PRS and ASD-PRS did not significantly differ between patients with SCZ and controls (**Supplementary Figure 1C**: ADHD-PRS, nominal *p* = 0.89; **Supplementary Figure 1D**: ASD-PRS, nominal *p* = 0.05).

### 3.2 Association between symptoms scale and PRSs

**Figure 2:**
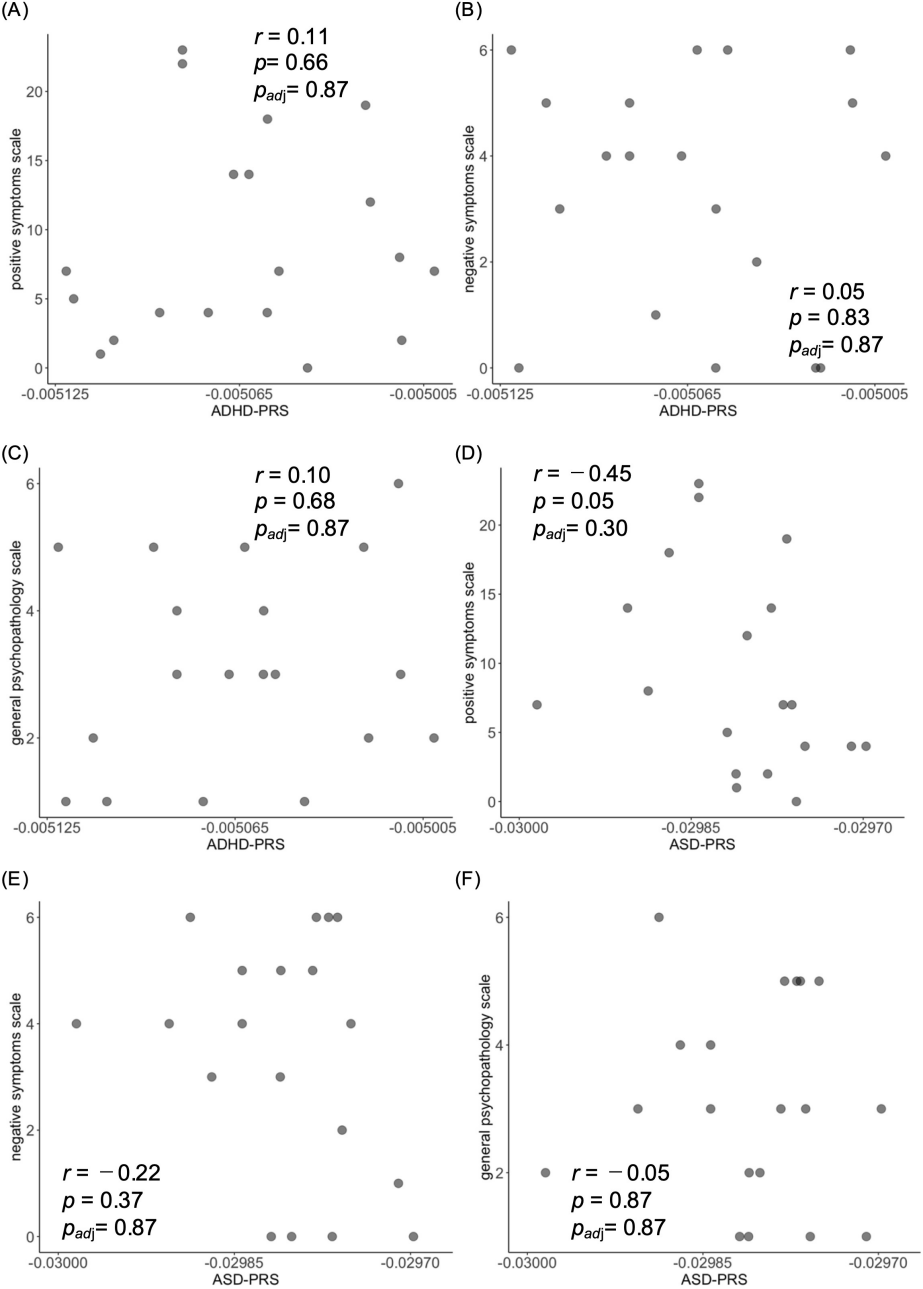
Correlation analyses between PRSs and DIBS. Scatter plots show a correlation between **(A)** ADHD-PRS and positive symptoms scale, **(B)** ADHD-PRS and negative symptoms scale, **(C)** ADHD-PRS and general psychopathology scale, **(D)** ASD-PRS and positive symptoms scale, **(E)** ASD-PRS and negative symptoms scale, and **(F)** ASD-PRS and general psychopathology scale. ADHD-PRS, polygenic risk score for ADHD; ASD-PRS, polygenic risk score for ASD; *p_adj_*, adjusted *p* value.

To investigate the relationship between SCZ symptoms and genetic risk for ADHD and ASD, we calculated the Spearman’s rank correlation coefficient between each DIBS subscale and the ADHD-PRS and ASD-PRS scores in 19 patients with SCZ. No significant correlations were observed between any DIBS subscale and either ADHD-PRS (**Figure 2A**: positive symptoms, **Figure 2B**: negative symptoms, **Figure 2C**: general psychopathology) or ASD-PRS (**Figure 2D**: positive symptoms, **Figure 2E**: negative symptoms, **Figure 2F**: general psychopathology).

### 3.3 Association between treatment resistant and PRSs

**Figure 3:**
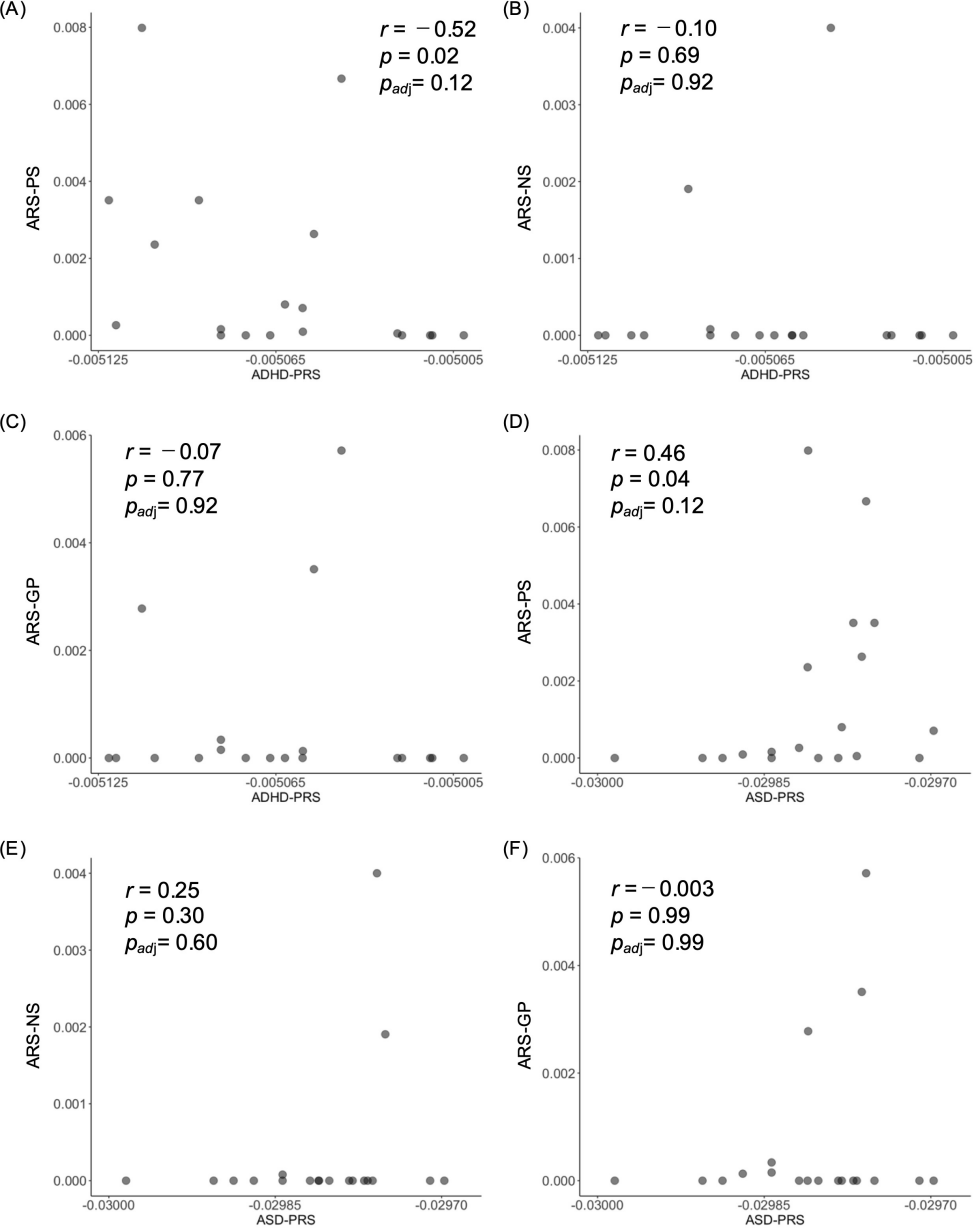
Correlation analyses between PRSs and ARS. Scatter plots show a correlation between **(A)** ADHD-PRS and ARS-PS, **(B)** ADHD-PRS and ARS-NS, **(C)** ADHD-PRS and ARS-GP, **(D)** ASD-PRS and ARS-PS, **(E)** ASD-PRS and ARS-NS, and **(F)** ASD-PRS and ARS-GS. ARS, antipsychotic responsiveness score; ARS-PS, ARS of positive symptoms; ARS-NS, ARS of negative symptoms; ARS-GP, ARS of general pathology; *p_adj_*, adjusted *p* value.

We then investigated the relationship between ARS and the genetic risk for ADHD and ASD using Spearman’s rank correlation. ARS-PS was negatively correlated with ADHD-PRS (**Figure 3A**: *r* = −0.52, nominal *p* = 0.02) and positively correlated with ASD-PRS (**Figure 3D**: *r* = 0.46, nominal *p* = 0.04) although these associations did not survive multiple testing correction. In contrast, ARS for negative symptoms (ARS-NS) and general psychopathology (ARS-GP) showed no significant correlation with either ADHD-PRS (**Figure 3B**: negative symptoms, **Figure 3C**: general psychopathology) or ASD-PRS (**Figure 3E**: negative symptoms, **Figure 3F**: general psychopathology).

### 3.4 Subgrouping patients based on PRSs

**Figure 4:**
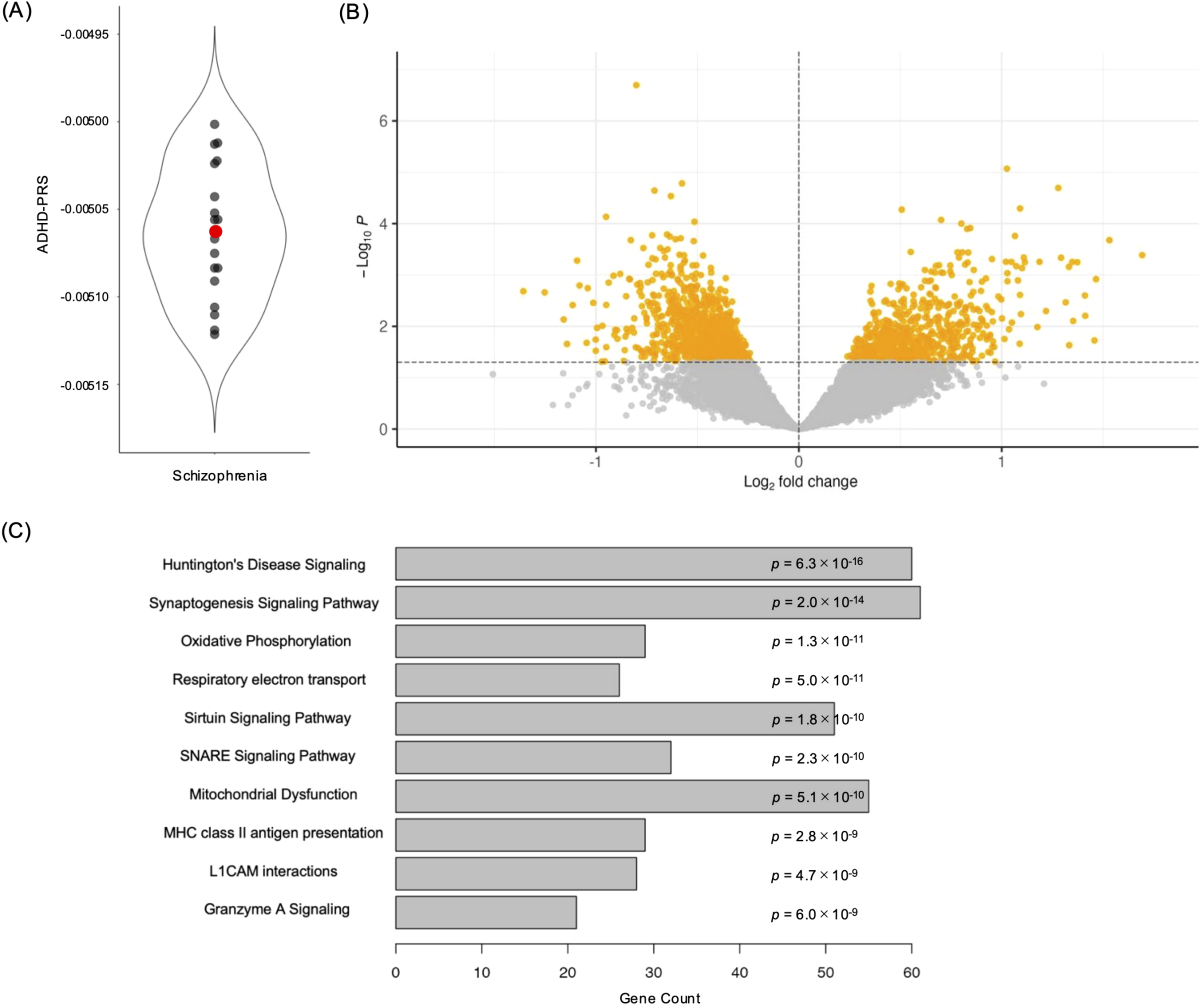
Gene expression analyses between subgroups. **(A)** The violin plot shows the distribution of PRS of patients with schizophrenia. The red mark represents the median. **(B)** Volcano plots showing differentially expressed genes (DEGs) between the subgroups. The DEGs (nominal *p* < 0.05) are highlighted in orange. **(C)** A bar plot showing the top 10 pathways that the DEGs were enriched in.

As ADHD has been more strongly associated with treatment resistance in SCZ than ASD, we hypothesized that the genetic risk of ADHD may influence ARS-PS. Therefore, we divided patients with SCZ into two groups: those with higher (high ADHD-PRS group: n = 9) and lower (low ADHD-PRS group: n = 10) ADHD-PRS values than the median (**Figure 4A**). The demographic data of patients in both subgroups are shown in **Table 1**.

### 3.5 Gene expression analyses

To investigate the molecular differences between the two subgroups, we compared mRNA expression levels in PFC as an exploratory analysis. Volcano plots are shown in **Figures 4B, C**. We identified 1,773 DEGs (nominal *p* < 0.05, **Supplementary Table 2**), suggesting that these genes may reflect molecular differences associated with variation in ARS. Among these, only *CHRNB2*, a gene coding the neuronal acetylcholine receptor (nAChR) subunit β2, remained significant after FDR correction (FDR < 0.05). No subject exhibited the extreme expression level of *CHRNB2* (**Supplementary Figure 2**). To examine the functions of the DEGs, we conducted canonical pathway analysis using IPA (**Figure 4D**, **Supplementary Table 3**). As the gene with FDR < 0.05 was only *CHRNB2*, we conducted pathway analyses on DEGs with nominal *p* < 0.05. These genes were enriched in pathways related to the neuronal system (“Huntington’s Disease Signaling,” “Synaptogenesis Signaling Pathway,” “SNARE Signaling Pathway,” and “L1CAM Interactions”), mitochondrial function (“Oxidative Phosphorylation,” “Respiratory Electron Transport,” “Sirtuin Signaling Pathway,” and “Mitochondrial Dysfunction”), and immune pathways (“MHC Class II Antigen Presentation” and “Granzyme A Signaling”). Pathway illustrations of “Synaptogenesis Signaling Pathway” and “Mitochondrial Dysfunction” are shown in **Supplementary Figure 3** and **Supplementary Figure 4**, respectively.

## 4 Discussion

In the current study, we adopted a novel approach to elucidate the effect of neurodevelopmental traits on SCZ, stratifying 19 patients with SCZ by PRSs for neurodevelopmental disorders. We investigated whether the genetic risks of ADHD and ASD were associated with the clinical symptom scales and ARS in SCZ. The ARS-PS showed a positive correlation with ASD-PRS and a negative correlation with ADHD-PRS despite the adjusted p-values not reaching statistical significance, suggesting that the genetic risks of neurodevelopmental disorders are associated with ARS. Additionally, stratification of patients with SCZ by ADHD-PRS and subsequent exploratory DEG analyses identified 1,773 DEGs (nominal *p* < 0.05), which were enriched in pathways related to neuronal and mitochondrial functions. These genes may be associated with treatment resistance linked to the genetic risks of neurodevelopmental disorders. Our novel approach elucidated that ADHD and ASD genetic risks may be associated with antipsychotic response profiles in SCZ, highlighting potentially related genes and pathways as exploratory findings.

There were no differences of ADHD-PRS and ASD-PRS between patients with SCZ and controls, which is often observed in the previous studies (40, 41). The ADHD-PRS and ASD-PRS did not correlate with any DIBS subscale, suggesting that the genetic risks for ADHD and ASD do not influence SCZ symptom severity. Consistently, ADHD-PRS and ASD-PRS are known to have limited effects on the severity of neurodevelopmental traits shared with SCZ (42, 43). Thus, it is challenging to explain the symptoms of neurodevelopmental disorders solely by genetic factors. Nevertheless, comorbidity of SCZ and ADHD is clinically important and is associated with poorer outcomes. For example, comorbid SCZ increases suicidal risk in patients with ADHD (44, 45), underscoring the importance of recognizing co-occurring psychiatric disorders in clinical practice.

In contrast, the ARS-PS was nominally associated with both ADHD-PRS and ASD-PRS. ASD-PRS positively correlated, whereas ADHD-PRS negatively correlated with treatment sensitivity to positive symptoms. The opposite effects of ADHD-PRS and ASD-PRS on ARS may stem from differences in the genetic architecture of the two disorders. Since ASD and ADHD frequently co-occur (46) and share overlapping genetic backgrounds (18), the opposite effects of their genetic risks on antipsychotic responsiveness are intriguing. Mattheisen et al. reported five loci differentiating ADHD and ASD, suggesting that some SNPs may have opposite effects on risk for the two disorders (47). These opposing effects highlight distinct pathophysiological pathways through which ADHD and ASD genetic risks may be associated with treatment outcomes. The data presented here suggest that the genetic risk of neurodevelopmental disorders appeared to be related to ARS-PS in patients with SCZ. These results indicate the hypothesis that genetic risk assessments for neurodevelopmental disorders could be an exploratory marker of treatment resistance of positive symptoms.

The finding that higher genetic risk for ADHD is associated with greater treatment resistance to positive symptoms raises a hypothesis that TRS and ADHD may share common pathophysiological features. Both ADHD and SCZ are thought to involve dopaminergic dysregulation. In ADHD, abnormalities in the dopaminergic system have been proposed to cause cognitive impairment and hyperactivity (48, 49). Meanwhile, positive symptoms in SCZ are thought to arise from excessive dopamine release (50, 51). Consistent with our findings, Cheng et al. reported a positive genetic correlation between TRS and ADHD (20). Additionally, Kuramitsu et al. found that patients with SCZ with higher genetic risk for ADHD than for ASD exhibited impaired working memory (19), a feature commonly linked to TRS (52). Those relationships between SCZ and ADHD lead to the speculative conjecture that pharmacological mechanisms might be shared. Notably, guanfacine, an α2-agonist used for ADHD treatment, reportedly improves a wide range of cognitive functions, including working memory, in patients with SCZ (53, 54). Additionally, clonidine, another α2-agonist, reportedly alleviates negative symptoms (55). Thus, the α2 receptor may represent a promising therapeutic target for SCZ and ADHD. Clozapine, the only effective treatment for TRS (56), has been shown to regulate dopamine release and normalize hyperactivity in a mouse model of dopamine dysregulation (57). Clozapine’s correcting effect on hyperactivity may suggest that its therapeutic action involves pathophysiological mechanisms shared between TRS and ADHD. Collectively, these results indirectly support the hypothesis that TRS and ADHD share genetic backgrounds and neurochemical mechanisms, including dopaminergic and other neurotransmission pathways targeted by clozapine.

Notably, a higher genetic risk for ASD was associated with greater ARS-PS, contrary to the effect of ADHD-PRS. This finding contrasts with previous studies suggesting that patients with TRS exhibit stronger ASD characteristics measured by autism spectrum quotient (AQ) than those in remission (21). Alternatively, our data suggest that such measures of autistic traits including AQ may fail to distinguish between autistic traits and psychotic symptoms. Autistic traits are life-long characteristics of ASD and related to ASD-PRS (58), while psychotic symptoms scales capture the fluctuating states of psychosis, which lack long-term stability, making these states often difficult to distinguish from autistic traits. For example, PAUSS, a common tool for assessing autistic traits in SCZ (59), lacks long-term stability and only captures treatable psychosis-related features (60). Our findings, therefore, support the hypothesis that the autistic traits observed in TRS are similar to, but distinct from, those of ASD. However, it should be noticed that the current study does not involve patients diagnosed as TRS. Therefore, further studies on patients with TRS are required to clarify the genetic relationship between TRS and neurodevelopmental disorders.

Unlike the positive symptom scale, no association was found between the genetic risk for neurodevelopmental disorders and ARS-NS or ARS-GP. This may be attributed to the limited efficacy of antipsychotics against negative symptoms and cognitive impairment. Indeed, among the 19 patients, only three showed improvements on the negative symptom scale, while six demonstrated improvements on the general psychopathology scale. To further investigate treatment resistance in these scales, a larger sample of patients with high ARS is required.

In this study, we divided patients into two subgroups based on ADHD-PRS. Patients in the high ADHD-PRS group had a higher genetic risk for ADHD and exhibited lower ARS values than those in the low-PRS group. An exploratory DEG analysis identified genes linked to neuropsychiatric disorders, among which, *CHRNB2* showed the strongest significance after multiple correction. *CHRNB2* participates in pathways such as “AMPK Signaling,” “Calcium Signaling,” and “Acetylcholine Receptor Signaling Pathway” (**Supplementary Table 3**). nAChR regulates dopamine release in the PFC, contributing to normal cognitive functioning (61, 62). The involvement of nAChRs, including the β2 subunit, in SCZ pathophysiology has been repeatedly reported. For example, several recent reviews have demonstrated that dysregulated cholinergic system in patients has been observed in SCZ (63, 64). Moreover, postmortem brain analyses have revealed altered expression of the α7 subunit in SCZ (65). The identification of *CHRNB2* in the present analysis suggests that AChR may represent a promising therapeutic target for patients exhibiting antipsychotic treatment resistance. Indeed, medications acting on α4β2 nAChRs have been shown to modulate the excitation/inhibition balance in human cortex (66). These findings collectively underscore the need for further research on AchR signaling in SCZ. Investigating *CHRNB2* gene expression levels using more rigorous methods, such as quantitative polymerase chain reaction (qPCR), may provide valuable insights into the pathophysiology of schizophrenia.

Canonical pathway analysis revealed that DEGs (nominal *p* < 0.05) were mainly enriched in neuronal, mitochondrial, and immune pathways. Enrichment of neuronal pathways (“Synaptogenesis Signaling Pathway,” “Signaling Pathway,” and “L1CAM interactions”) suggests that disrupted synaptic structure and function contribute to the pathophysiology and treatment responsiveness of SCZ. Accumulating evidence from postmortem brains and induced pluripotent stem cells (iPSCs) support synaptic dysfunction in SCZ (50, 67–69). Taken together, these findings may indicate that synaptic dysfunction contributes to cognitive impairment and negative symptoms (50). Patients with TRS also exhibit disrupted brain connectivity patterns (70, 71). These findings may raise the hypothesis that abnormalities in synaptic structure and connectivity potentially contribute to treatment resistance in SCZ. Further studies on synaptic structure and connectivity in SCZ may elucidate the molecular basis of treatment resistance.

Mitochondrial pathways (“Oxidative Phosphorylation,” “Respiratory Electron Transport,” “Sirtuin Signaling Pathway,” and “Mitochondrial Dysfunction”) were also the pathway that DEGs (nominal *p* < 0.05) enriched in. Mitochondrial dysfunction has been repeatedly implicated in psychiatric disorders, including SCZ and ADHD (72–74). Moreover, mitochondrial dysfunction may relate to clinical symptom severity (75). Our enrichment analyses suggest that mitochondrial dysfunction may appear to be related to antipsychotic responsiveness in patients with SCZ. Our exploratory findings indicate the possibility that mitochondrial dysfunction may play a pivotal role in both the etiology and treatment responsiveness of SCZ.

Finally, DEGs (nominal *p* < 0.05) were enriched in immune pathways (“MHC Class II Antigen Presentation” and “Granzyme A Signaling”). Dysregulation of immune pathways has been repeatedly implicated in SCZ, with strong evidence implicating MHC-related mechanisms. For example, proteomic studies of the dorsolateral PFC have identified altered expression of MHC-related proteins in SCZ (76). Additionally, granzyme A, which exhibited altered expression in our dataset, has been previously reported to show reduced blood expression in untreated patients with SCZ (77). These preliminary findings may support the hypothesis that immune molecules, including MHC class II and granzyme A, play crucial roles in SCZ pathophysiology.

This study has some limitations. First, the sample size was relatively small because detailed information on SNPs, transcriptomes, and clinical data was required. To the best of our knowledge, there has been no public data repository providing that information on postmortem brains from patients with SCZ. The low statistical power due to the limited sample size raises the possibility that the current results may be due to statistical instability rather than true biological effects, including the opposite effects of ASD-PRS and ADHD-PRS on ARS, which did not reach significance after the multiple testing correction, and results of gene expression and pathway analyses. Especially, the current DEG detection analyses included only sex and age at death as covariates to avoid overfitting, which may introduce potential bias and low statistical power. Pathway analyses may also leave the possibility of false-positive because DEGs which does not survive the multiple testing correction. Ideally, validation experiments using qPCR on independent cohort should be conducted. Second, the discovery GWASs used to calculate ADHD- and ASD-PRS were derived from populations of European ancestry because no large-scale GWAS summary statistics from East Asian populations (including Japanese) with ADHD and ASD have been available to date, which may limit generalizability and potentially decrease robustness (78). Replication studies on genetically matched subjects are required. Third, ARS were assessed retrospectively following the patients’ death, which may potentially result in the biased findings because clinical information close to death may not accurately reflect longitudinal treatment response to exclude the postmortem nature of the data. Thus, further studies investigating longitudinal treatment response are required. Despite of those limitations, exploratory findings between PRSs and ARS in the current study indicated that stratifying patients with SCZ based on PRSs for neurodevelopmental disorders might be a promising approach to partially predict treatment resistance. Our findings warrant future studies with larger, ancestrally diverse samples to elucidate the robust associations between neurodevelopmental traits and SCZ pathophysiology.

In conclusion, the genetic risks for ADHD and ASD may be associated with the antipsychotic responsiveness of patients with SCZ. Furthermore, in an exploratory gene expression analysis, we identified DEGs (nominal *p* < 0.05) that may play critical roles in determining this responsiveness. Our exploratory findings provide new insights into the pathophysiology and therapeutic strategies for patients exhibiting antipsychotic treatment resistance.

## Data Availability

The data analyzed in this study is subject to the following licenses/restrictions: The datasets presented in this article are not readily available because of privacy restrictions. Requests to access the datasets should be directed to the corresponding author/s. Requests to access these datasets should be directed to Atsuko Nagaoka, M.D., Ph.D. atsuko.nagaoka.b5@tohoku.ac.jp.
