# Supplementary material for: Genetic risk for neurodevelopmental disorders as a potential factor affecting antipsychotic responsiveness in schizophrenia: a postmortem brain study
Source: Front Psychiatry. 2026 Apr 27;17:1812198. doi: 10.3389/fpsyt.2026.1812198 (PMC13158216; doi:10.3389/fpsyt.2026.1812198)
Supplement: Supplementary file 2 [file SupplementaryFile1.pdf]

Supplementary Material

1 Supplementary Figures

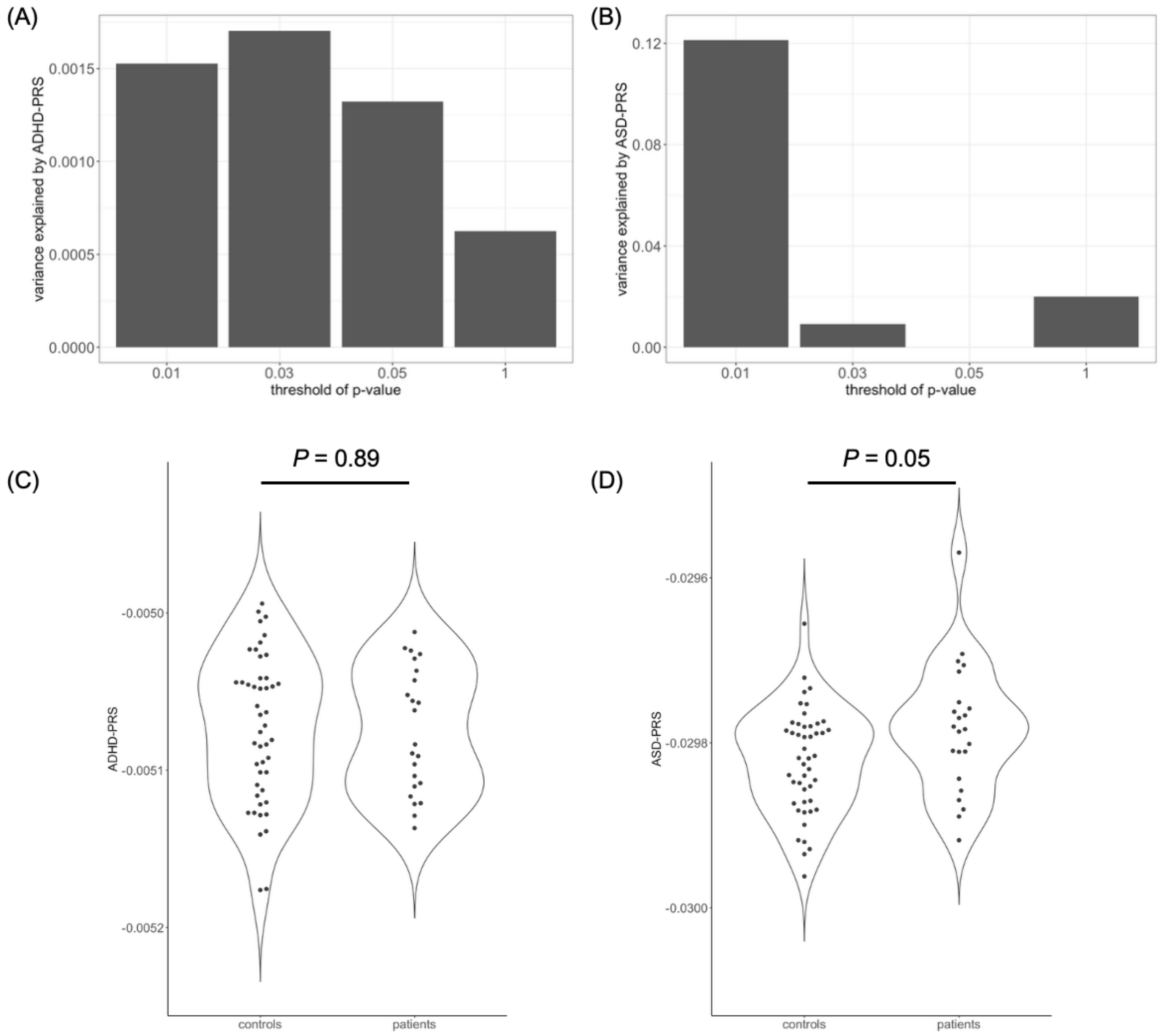

Supplementary Figure S1. Calculation of ADHD-PRS and ASD-PRS. Bar plots show a variance in the risk for schizophrenia explained by (**A**) ADHD-PRS and (**B**) ASD-PRS with different p-value thresholds. Violin plots show (**C**) ADHD-PRS and (**D**) ASD-PRS in controls and patients with schizophrenia. ADHD-PRS, polygenic risk score for ADHD; ASD-PRS, polygenic risk score for ASD.

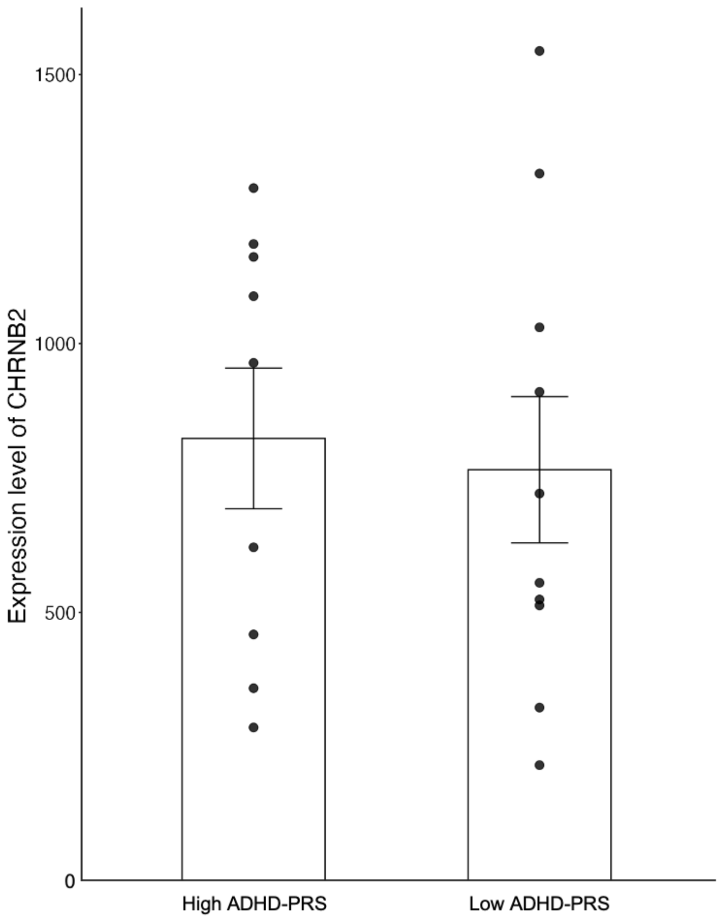

Supplementary Figure S2. The expression level of *CHRNB2* of patients with high and low ADHD-PRS. Error bar indicates a standard error.

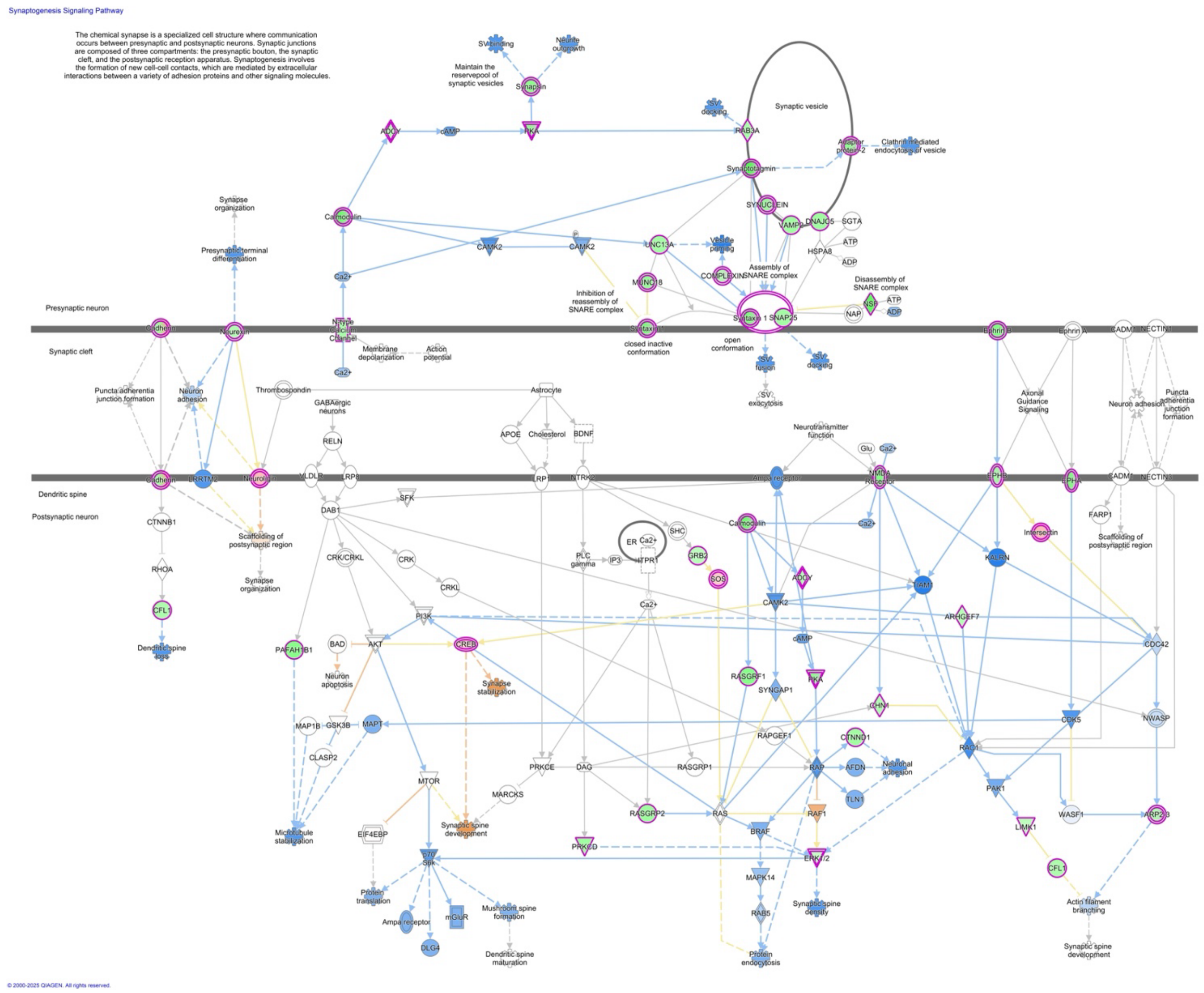

Supplementary Figure S3. An Ingenuity Pathway Analysis illustration showing Downregulation of the Synaptogenesis Signaling Pathway. A canonical pathway of the "Synaptogenesis Signaling Pathway" is downregulated in high ADHD-PRS patients with schizophrenia. Nodes contain information on DNA, RNA, and proteins. Nodes with a pink border represent those that are differentially expressed at the mRNA level between bipolar I and II disorder. Red-filled nodes represent upregulated expression, while green-filled nodes represent downregulated expression. Nodes noted in orange were predicted to be upregulated in diseases or biological functions, which may include an increase in mRNA expression levels or activation of proteins. Conversely, nodes noted in blue were predicted to be downregulated in diseases or biological functions. The orange line represents the predicted activation relationship between the upstream node and the downstream node, consistently supported by IPA knowledge. The blue line represents the predicted inhibition relationship. The yellow line represents an inconsistent relationship. The gray line represents no prediction information. The network was generated using QIAGEN IPA. For more detailed information, please refer to https://qiagen.my.salesforce-sites.com/KnowledgeBase/KnowledgeNavigatorPage?id=kA41i000000L5rTCAS&categoryName=IPA.

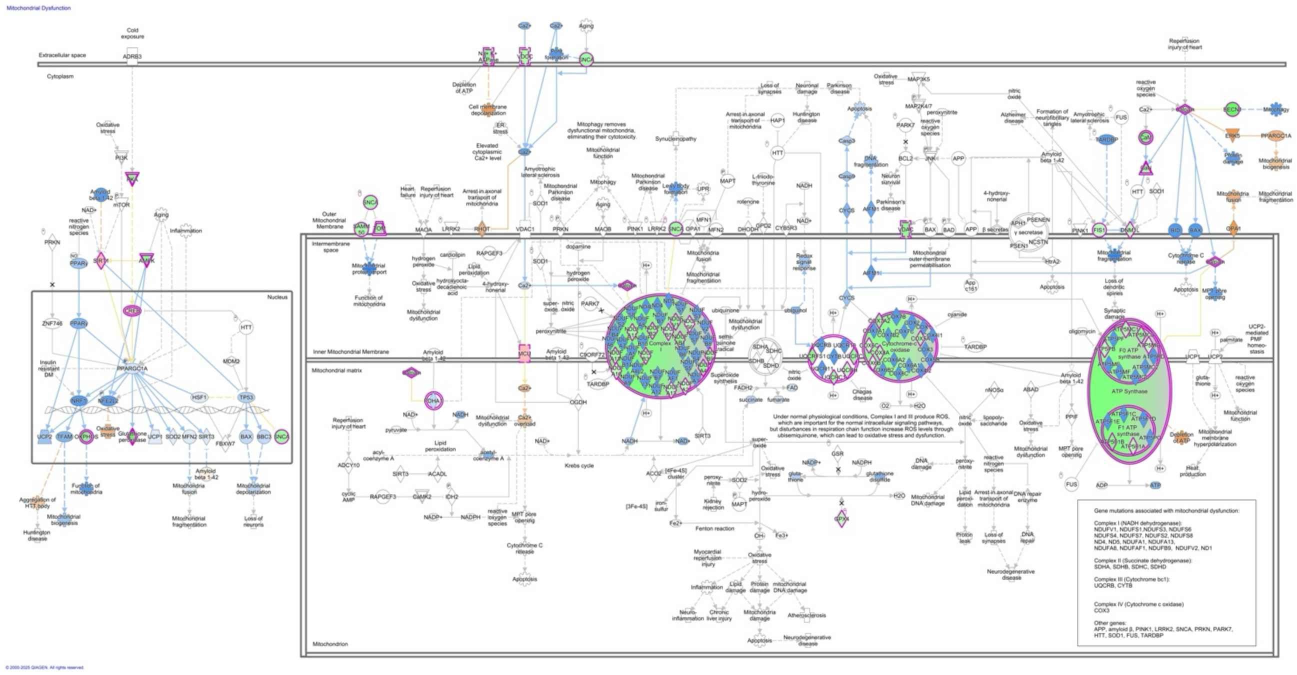

Supplementary Figure S4. An Ingenuity Pathway Analysis illustration showing Downregulation of the Mitochondrial Dysfunction pathway. A canonical pathway of the "Mitochondrial Dysfunction" is downregulated in high ADHD-PRS patients with schizophrenia. Nodes contain information on DNA, RNA, and proteins. Nodes with a pink border represent those that are differentially expressed at the mRNA level between bipolar I and II disorder. Red-filled nodes represent upregulated expression, while green-filled nodes represent downregulated expression. Nodes noted in orange were predicted to be upregulated in diseases or biological functions, which may include an increase in mRNA expression levels or activation of proteins. Conversely, nodes noted in blue were predicted to be downregulated in diseases or biological functions. The orange line represents the predicted activation relationship between the upstream node and the downstream node, consistently supported by IPA knowledge. The blue line represents the predicted inhibition relationship. The yellow line represents an inconsistent relationship. The gray line represents no prediction information. The network was generated using QIAGEN IPA. For more detailed information, please refer to https://qiagen.my.salesforce-sites.com/KnowledgeBase/KnowledgeNavigatorPage?id=kA41i000000L5rTCAS&categoryName=IPA.
